# Non-uniform Evolving Hypergraphs and Weighted Evolving Hypergraphs

**DOI:** 10.1038/srep36648

**Published:** 2016-11-15

**Authors:** Jin-Li Guo, Xin-Yun Zhu, Qi Suo, Jeffrey Forrest

**Affiliations:** 1 Business School, University of Shanghai for Science and Technology, Shanghai 200093, PR China; 2 School of Business, Slippery Rock University, Slippery Rock, PA 16057, USA

## Abstract

Firstly, this paper proposes a non-uniform evolving hypergraph model with nonlinear preferential attachment and an attractiveness. This model allows nodes to arrive in batches according to a Poisson process and to form hyperedges with existing batches of nodes. Both the number of arriving nodes and that of chosen existing nodes are random variables so that the size of each hyperedge is non-uniform. This paper establishes the characteristic equation of hyperdegrees, calculates changes in the hyperdegree of each node, and obtains the stationary average hyperdegree distribution of the model by employing the Poisson process theory and the characteristic equation. Secondly, this paper constructs a model for weighted evolving hypergraphs that couples the establishment of new hyperedges, nodes and the dynamical evolution of the weights. Furthermore, what is obtained are respectively the stationary average hyperdegree and hyperstrength distributions by using the hyperdegree distribution of the established unweighted model above so that the weighted evolving hypergraph exhibits a scale-free behavior for both hyperdegree and hyperstrength distributions.

Complex networks can be used to describe and understand a variety of real-life systems, be they complex interacting systems or the microscopic nature of space-time. In 1998, the small-world characteristic of complex networks was first found by Watts and Strogatz^1^. Then in 1999, the emergence of scaling in complex networks was discovered by Barabási and Albert^2^. In addition, growth and preferential attachment were regarded as two basic mechanisms of complex networks. Different kinds of complex networks have attracted the attention of scholars in recent decades. A complex network is a graph with non-trivial topological features that do not occur in simple graphs such as lattices or random graphs but often occur in modelling real-life systems. Since late 20th century, studies of complex networks have been undertaken in many disciplines, including mathematics, physics, computer science, biology, social science, and economics. Complex network models have been used to study different networks in real-life world, such as protein-protein interaction networks^3^, food chain networks^4^, transportation networks^5^, large-scale grid networks, economic networks, and social networks^6^,^7^. Throughout the past ten years, scientists have constructed various models to describe the characteristics of complex networks and proposed many analysis methods to model and to optimize networks that exist in real-life^8^. Theoretical studies on complex networks are now making a transition into the more organized systematic way.

However, it is still hard to depict some real-life systems by using the concept of complex networks. Due to the complication involved with real-world systems, simple graphs are no longer suitable for depicting networks with different kinds of nodes. The energy-supply network^9^ can be seen as a part of a larger system, in which interdependent networks with different structures and functionalities coexist, interact, and coevolve. Nicosia *et al*.^10^ proposed a modelling framework for growing multiplexes where a node can belong to different networks. Some nodes in real-life networks may exhibit two or more properties, while nodes in complex networks might maintain their homogeneity. For example, the nodes in a supply chain^11^ belong to different categories including manufacturers, consumers, and so on. The nodes in a grid network also share different characteristics, including power substations, consumers, etc. Consequently, simple graphs are not adequate to represent such systems. On the other hand, these graphs also not suitable for any network with one edge containing more than two nodes, either. For example, the scientific collaboration network^12^ may not be suitable for complex networks to represent, because there are usually more than two authors on one paper. Ecological networks are normally represented by competition graphs in which only two species competing for their common prey can be investigated. The concept of complex networks fails to provide the information about the whole groups of species with a particular prey. In these cases, using simple graphs to represent complex networks does not provide a complete description of the real-world system. A natural way of representing these systems is to use a generalization of graphs known as hypergraphs^13^,^14^. The first application of hypergraphs for representing social networks appeared in 1981 as reported by Seidman. The competition hypergraph was proposed to develop a more complete description in which nodes denote species and hyperedges sets of species having the same prey. In a chemical reaction network, nodes and hyperedges are defined respectively as chemical compounds and reactions. Because a chemical reaction represents a process that involves a set of chemical compounds, substrates, and more than one product, the hypergraph representation is indispensable^15^. In order to consider multi-protein complexes, a hypergraph is used to represent a protein network, in which nodes and hyperedges represent proteins and complexes, respectively^16^. Although some real-life systems have been represented by bipartite graphs or tripartite graphs, their properties may be different when depicted by hypergraphs. In this paper, we will extend these concepts for complex networks that are represented by hypergraphs.

The concept of hypergraphs offers a new tool for depicting real-life systems, and has been gaining more attention in recent years. Boccaletti *et al*.^17^ believed that a hypergraph can be regarded as a special case of a multilayered network. Park *et al*.^18^ applied the concept of hypergraphs in a cell bio-molecular system, and found that the hypergraph structure was very helpful in discovering the building blocks of the higher-order interactions of multiple variables. In addition, they applied the hypergraph model in the analysis of microarray data for cancer diagnosis. Akram and Dudek^19^ developed a different application of hypergraphs. They combined intuitionistic fuzzy theory with the hypergraph concept and defined several intuitionistic fuzzy structures, which are more flexible than the classic models. Bootkrajang *et al*.^20^ built a model of associative memory based on an undirected hypergraph of weighted edges. Elena and Vladimir^21^ used the hypergraph theory to study molecular structures of compounds and distinguished these structures by their different topological indices. Johnson^22^ introduced the definition of hypernetworks. That is, the concept of hypernetworks is a natural multidimensional generalization of networks and represents n-ary relations by simplices with n vertices.

Since a hypergraph is a natural extension of a graph, the concept of complex network can be extended to that of evolving hypergraph; and evolving hypergraphs refer to such hypergraphs that represent complex systems^23^. Recently, various scholars have studied the topological properties and models of evolving hypergraphs. Estrada and Rodríguez-Velázquez^23^ studied the subgraph centrality and the clustering coefficient. Wang *et al*.^24^ proposed a dynamic evolving model according to uniform growth and preferential attachment mechanisms, in which a new batch of nodes together with one existing node formed one hyperedge, and gradually formed the final network. Hu *et al*.^25^ proposed another dynamic model. The growth and preferential attachment mechanisms of the model are the same as those of Wang’s model, but at each time step there is only one newly added node. Guo and Zhu^26^ developed a unified model and the model can be degenerated to the original model as proposed by Barabási and Albert^2^. Guo and Suo^27^ also developed a model with the brand effect and competitiveness. Although a few of models in evolving hypergraphs have been proposed based on uniform growth, there are no evolving models considering the non-uniform characteristics, which may have a huge potential for applications in the study of real-life systems.

The afore-mentioned models are all unweighted evolving hypergraphs. The purpose of this paper is to extend the concept of evolving hypergraphs by combining the characteristics of non-uniformity and weight of hyperedges. Firstly, we propose a non-uniform model with nonlinear preferential attachment and an attractiveness, and establish the characteristic equation of hyperdegrees and the stationary average hyperdegree distribution by using the Poisson process theory and the characteristic equation. Our theoretical analysis is in good agreement with the simulation results. Secondly, we propose a weighted model to better describe real-life systems. It is found that the hyperdegree and hyperstrength distributions of our weighted model can be directly obtained from the unweighted model with attractiveness.

## Non-Uniform Evolving Hypergraphs with Attractiveness

The evolving hypergraphs in the existing literatures are all uniform. That is, each hyperedge connects exactly k nodes. However, at each time step the number of new nodes entering into the network or previously existing nodes selected may not be the same. For instance, in the scientific collaboration networks, the nodes contained in a hyperedge, which is used to describe all the authors of a paper, are usually uncertain. In Wechat networks, the number of nodes contained in a group of friends is also uncertain. In these cases, simple uniform evolving hypergraphs cannot provide the complete information of the real-life systems of concern. For convenience, for the definition of evolving hypergraphs, please consult with ref. 23. Let 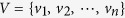 be a finite set, and 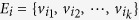 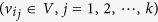, 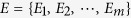 a family of subsets of *V*. The pair *H* = (*V, E*) is called a hypergraph. An element in *V* is called a node, and 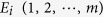 is called a hyperedge. In a hypergraph, two nodes are said to be adjacent if there is a hyperedge that contains both of these nodes. Two hyperedges are said to be adjacent if their intersection is not empty. If the cardinality |*V|* of *V* and the cardinality |*E*| of *E* are finite, respectively, then *H* is said to be a finite hypergraph. If 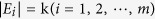, then *H* = (*V, E*) is a *k*-uniform hypergraph. Based on these definitions, we can introduce the following mathematical definition of the evolving hypergraph. Suppose that 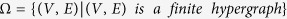 and *G* a map from 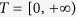 into Ω. For any given 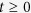, 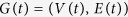 is a finite hypergraph. The index *t* is often interpreted as time. An evolving hypergraph 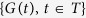 is a collection of hypergraphs that represent complex systems. The hyperdegree of *v*_*i*_ is defined as the number of hyperedges that connect to node *v*_*i*_.

**Figure 1:**
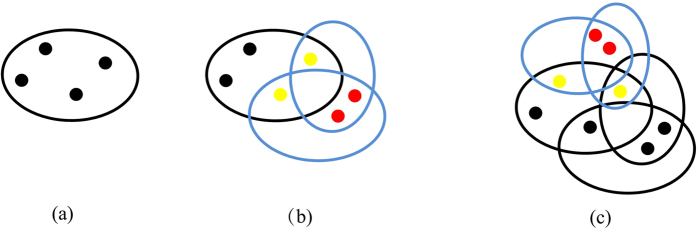
Schematic illustration of the evolving process of the non-uniform model. (**a**) is the initial network, (**b**) is second time step, (**c**) is third time step. A red disk denotes a new node. A yellow disk denotes an existing node selected to form a hyperedge with a probability proportional to its hyperdegree and attractiveness. A blue ellipse denotes a new hyperedge.

A non-uniform model with an attractiveness is defined as follows: (i) The network starts from an initial seed of *m*_0_ nodes and a hyperedge containing *m*_0_ nodes. Suppose that new node batches arrive at the system according to a Poisson process *N*(*t*) with rate *λ*. Each node entering the network is tagged with its own attractiveness *a*. At time *t*, 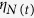 and 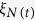 are positive integers that are taken from the given probability density functions 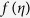 and 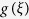, respectively. (ii) When a new batch of 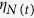 nodes is added to the network at time *t*, these 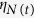 new nodes and 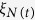 previously existing nodes are encircled by a new hyperedge, totally *m (mm*_2_ ≤ *m*_0_) new hyperedges are constructed with no repetitive hyperedges. The probability that a new node will connect to the *j*th node of the *i*th batch, is proportional to a sublinear function of the hyperdegree 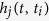 and attractiveness *a* such that


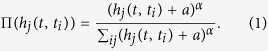


where *t*_*i*_ denotes the time when the *i*th batch of nodes enters into the network, that is to say, the birth time of the *i*th batch of nodes is *t*_*i*_. The symbol 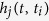 denotes the hyperdegree of the *j*th node of the *i*th batch. And *α* (0 ≤ *α* < 1) is a constant, 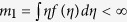, 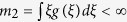. The evolving process of the model is shown in Fig. 1.

By a preferential attachment mechanism, it means that the higher the node hyperdegree is, the more probability it will be connected. The preferential probability of the old nodes will be higher than that of new nodes, namely, the phenomenon that “the rich get richer.” For example, in the scientific collaboration networks, nodes and hyeredges denote the authors and the papers they have written, respectively. The node with higher hyperdegree might be a famous author in his field who has written many papers. When new authors enter into the network, they tend to cooperate with those famous authors in order to gain their own fame.

Supposing that 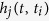 is a continuous real variable which is proportional to probability 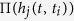. Then, by using techniques of continuous technique, it can be seen that 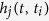 satisfies the following dynamical equation.


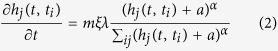


where 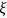 is a random variable taken from the probability density function 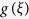.

The symbol *N*(*t*) denotes the total number of batches of nodes at time *t*. By employing the Poisson process theory, we have 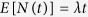. Let


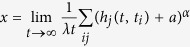


then we have,


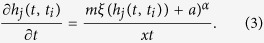


The solution of this equation, with the initial condition that a node in the *i*th batch at its birth time satisfies 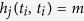, is


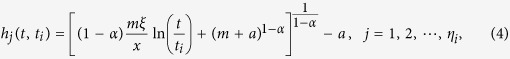


where *x* satisfies the following equation


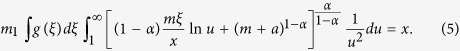


The Eq. (5) is called the characteristic equation of hyperdegree of the model.

From Eq. (4), it follows that


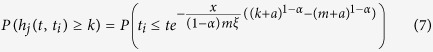


Notice that the arrival process of node batches is a Poisson process having rate *λ*. Therefore time *t*_*i*_ follows a gamma distribution with parameter(*i, λ*), and thus we have


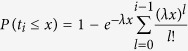


From Eq. (7), we have


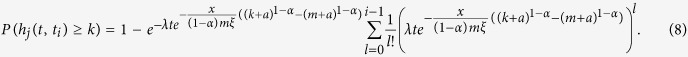


From Eq. (8), we obtain


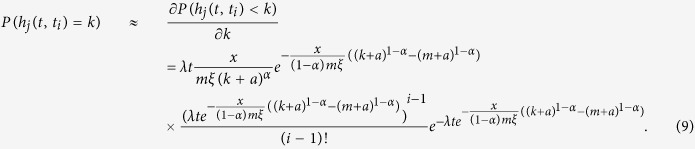


From Eq. (9), it follows that the stationary average hyperdegree distribution is:


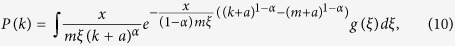


where *x* is a common positive solution of Eq. (5). This result shows that the hyperdegree distribution not only depends on the exponent *α* of the nonlinear preferential attachment, but also relates with the distribution of the number of chosen existing nodes.

When *α* = 1, since 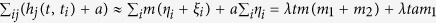


we can directly obtain


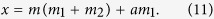


Substituting Eq. (11) into Eq. (10) yields,


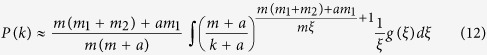


When *α* = 1, 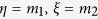, from Eq. (12), we have


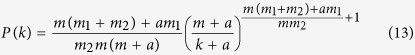


Equation (13) exhibits the scale-free property of the evolving hypergraph, and the hyperdegree distribution behaves as 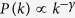, where


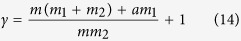


If 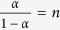, that is, 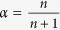, then the characteristic equation Eq. (5) is reduced to,


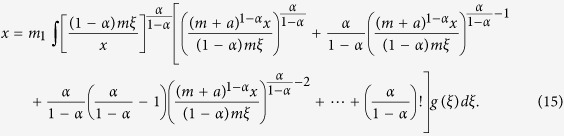


where *n* is a nonnegative integer.

If *α* = 0, from Eq. (15), we have *x* = *m*_1_. From Eq. (10), we obtain


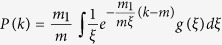


If *α* = 1/2, from Eq. (15), we obtain


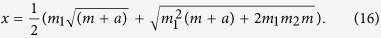


Substituting Eq. (16) into Eq. (10) yields,


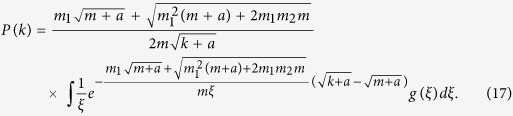


If *α* = 2/3, from Eq. (15), we obtain the characteristic equation of hyperdegree of the model


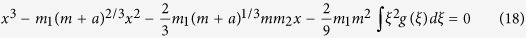


**Figure 2:**
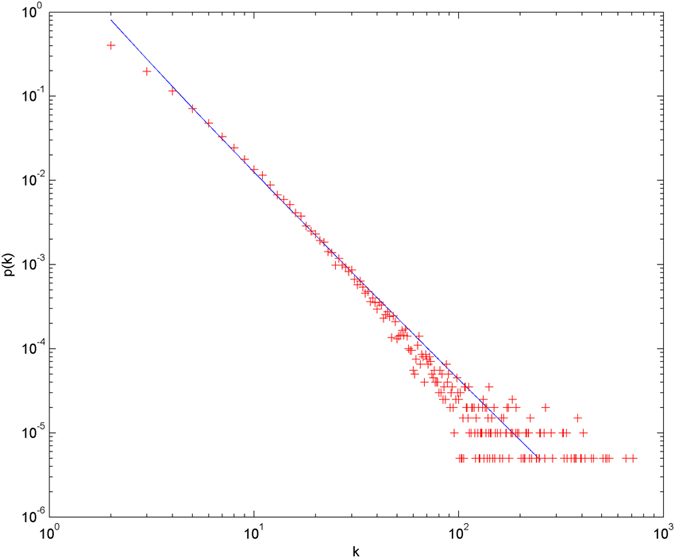
The simulation of the non-uniform model. *α* = 1, 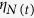 is randomly selected from 1 ~ 3, 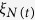 is randomly selected from 1 ~ 5. + denotes the simulation result, the line denotes the theoretical prediction.

**Figure 6:**
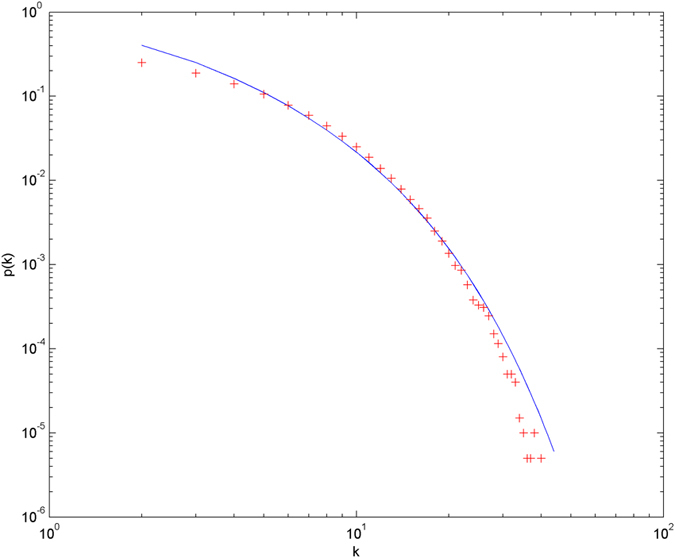
The simulation of the non-uniform model. *α* = 0, 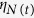 is randomly selected from 1 ~ 3, 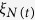 is randomly selected from 1 ~ 5. + denotes the simulation result, the line denotes the theoretical prediction.

In the following simulations, the parameters are set as follows: the number of initial nodes *m*_0_ = 10, the number of hyperedges *m* = 2, the attractiveness *a* = 1. The simulations are performed with scale of *N* = 100000 (the total number of nodes is 100000), and each simulation result is obtained by averaging over 30 independent runs. The simulation results are shown from Figs 2 and 6 in double-logarithmic axis. As the figures show, the theoretical prediction of the hyperdegree distribution is in good agreement with the simulation results.

## Weighted Evolving Hypergraphs

The mathematical definition of weighted evolving hypergraphs is given as follows. The concept of weighted hypergraphs generalizes that of simple weighted graphs by allowing for edges of higher cardinality. Formally, we define a weighted hypergraph as a triple 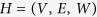, where 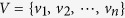, 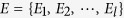 (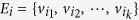 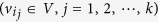), 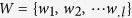 (*w* is a map from *E*_*e*_ into the set *R* of real numbers, denoted by 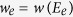) are the sets of nodes, edges and weights, respectively. In the weighted hypergraph, two nodes are said to be adjacent if there is a hyperedge that contains both of these nodes. Two hyperedges are said to be adjacent if their intersection is not empty. If the cardinality 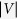 of *V* and the cardinality 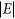 of *E* are finite, respectively, *H* is said to be a finite weighted hypergraph. If 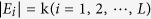, then 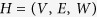 is a *k*-uniform weighted hypergraph. Suppose 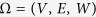 is a finite weighted hypergraph and *G* is a map from 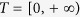 into Ω; for any given *t* ≥ 0, 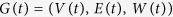 is a finite weighted hypergraph. The index *t* is often interpreted as time. A weighted evolving hypergraph 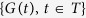 is a collection of weighted hypergraphs. The hyperdegree of *v*_*i*_ is defined as the number of hyperedges that connect to node *v*_*i*_. For the hyperedges that are connected to *v*_*i*_, the sum of their hyperedge weights is called the hyperstrength of *v*_*i*_.

**Figure 7:**
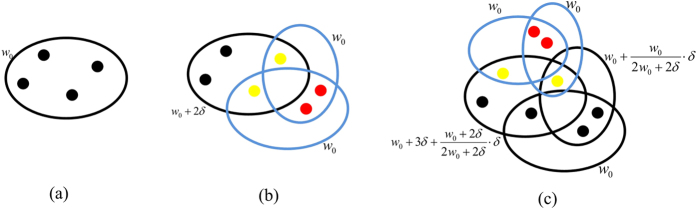
Schematic illustration of the evolving process of the weighted model. (**a**) is the initial network, (**b**) is the second time step, (**c**) is the third time step. The weight of each new hyperedge is *w*_0_.

In the BBV (Barrat-Barthelemy-Vespignani) model^28^,^29^ of complex networks, nodes enter into the network one by one, and the edges formed by one new added node and one old node. This model plays an important role in complex networks. However, it can only represent relations between a pair of nodes. However, edges in many real-world problems should involve information such as cooperation, trade or interaction among more than two actors. In addition, the interaction strength (the weight) of the edge characterizes real networks. For instance, the scientific collaboration network^4^ can be viewed as a weighted evolving hypergraph, where the weight of hyperedges should be the number of papers cooperated by co-authors. In airline networks, the weight of edges is used to represent passenger flow volume. Similarly, in trade networks the weight of edges is used to represent total trade between countries. In transportation networks, metro lines are always added with more than one node at each time step. These networks are different from simple weighted networks. Thus, this paper proposes a model of weighted evolving hypergraph to describe the weighted hyperedge growth caused by batches of newly added nodes. The theoretical analysis result and simulations are obtained. The definition of weighted models is based on two coupled mechanisms: the topological growth and the weights’ dynamics. The weighted model is defined as follows:

(i) *Growth*: The network starts from an initial seed of *m*_0_ nodes and a hyperedge containing *m*_0_ nodes, and the hyperedge is assigned with weight *w*_0_. Suppose that nodes arrive at the system according to a Poisson process with rate *λ*. If *m*_1_ new nodes arrive to the network at time *t*, one new hyperedge is formed by these new nodes and *m*_2_ previously existing nodes, totally *m (mm*_2_ ≤ *m*_0_) new hyperedges are constructed with no repetitive hyperedges.

(ii) *Hyperstrength driven attachment*: The new batch of nodes preferentially chooses nodes with larger hyperstrength, i.e., the probability that the new batch nodes will connect to previously existing node *v*_*ij*_ (the symbol *v*_*ij*_ denotes the *j*th node of the *i*th batch) is proportional to the hyperstrength 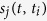 of node *v*_*ij*_, such that


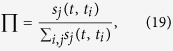


where 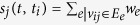 is the hyperstrength of *v*_*ij*_. (iii) *Weights’ dynamics*: The weight of each new hyperedge is initially set to a given value w_0_. A new hyperedge of node *v*_*ij*_ will trigger only local rearrangements of weights on the previously existing neighbors 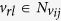, where 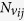 represents the neighbors of *v*_*ij*_, according to the simple rule


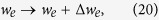


where 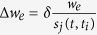, 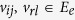, *δ* = *const* is defined as updating coefficient. The evolving process of the model is shown in Fig. 7. 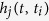 denotes the hyperdegree of node *v*_*ij*_. When a new batch arrive at the system, an already present node *v*_*ij*_ can be affected in two ways: (a) It is chosen with probability Eq. (19) to be connected to the batch of new nodes, then its hyperdegree increases by 1, and its hyperstrength by *w*_0_ + *δ*. (b) One of its neighbors 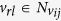 is chosen to be connected to the batch of new nodes, then the hyperdegree of *v*_*ij*_ is not modified, but *w*_*e*_ is increased according to the rule in Eq. (20), and thus *s*_*ij*_ is increased by 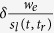. This dynamical process is modulated by the respective occurrence probabilities 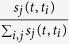 and is thus described by the following evolution equations for 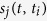 and 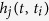:


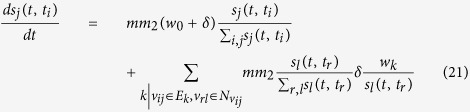



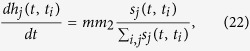


Since 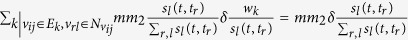, the following is obtained


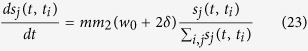


Substituting Eq. (22) into Eq. (23) yields


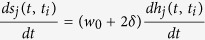


Since node 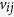 arrives at the system at time *t*_*i*_, we have 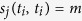 and 
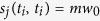
. Then integrating the equation above from *t*_*i*_ to *t* produces


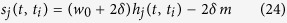


and probability Eq. (19) is modified as follows:


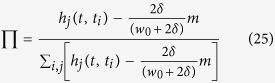


By comparing Eqs (25) and (1), it can be inferred that the attractiveness of the evolving hypergraph model is as follows,


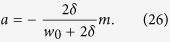


The probability of the preferential attachment in this model can be modified as 
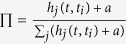
, which is in accordance with that of the model with an attractiveness. Substituting Eq. (26) into Eq. (13) yields the stationary average hyperedegree distribution of the weighted model:


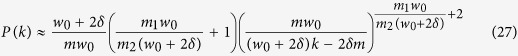


Moreover, from Eq. (27), it follows that the hyperdegree distribution behaves as 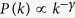, where


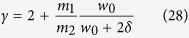


Therefore, the hyperdegree distribution of the weighted model can be obtained directly from the results of the model with an attractiveness.

When 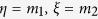, from Eqs (8), (24) and (26), we have


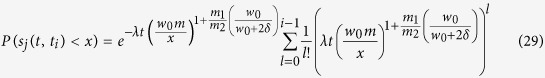


Hence, the density function of *s*_*j*_(*t, t*_*i*_) is


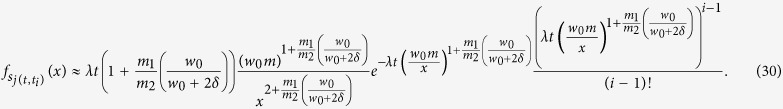


So, the density function *f*(*x*) of the stationary average hyperstrength distribution can be deduced from Eq. (30) as follows:


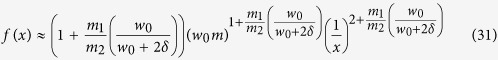


Evidently, from Eq. (31), we know that the stationary average hyperstrength distribution of the weighted model is a power-law distribution.

**Figure 8:**
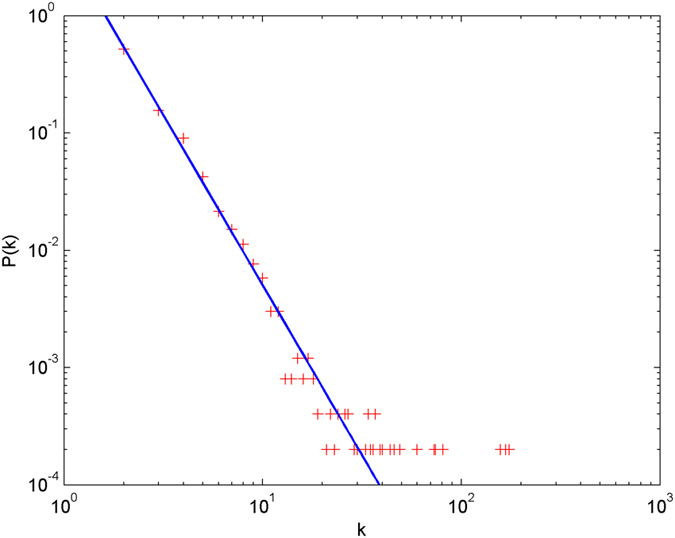
The simulation of the weighted model for node hyperdegree with *m*_1_ = 2, *m*_2_ = 1, *m* = 2, *w*_0_ = 1, *δ* = 1. + denotes the simulation result, the line denotes the theoretical prediction.

**Figure 9:**
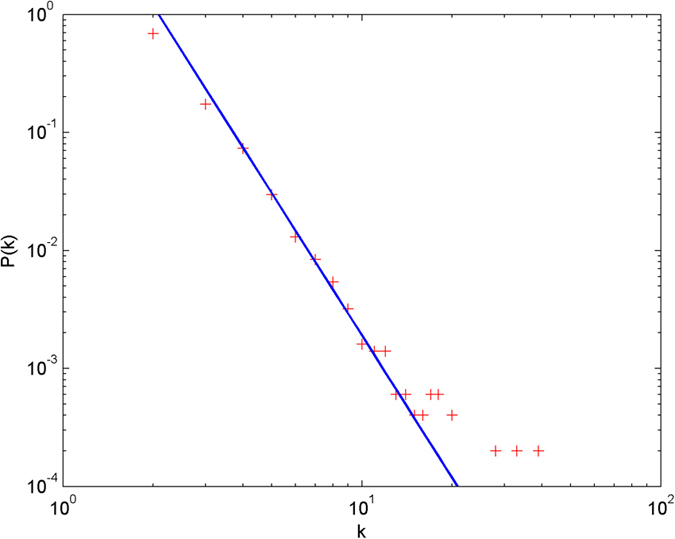
The simulation of the weighted model for node hyperdegree with *m*_1_ = 3, *m*_2_ = 1, *m* = 2, *w*_0_ = 5, *δ* = 1. + denotes the simulation result, the line denotes the theoretical prediction.

**Figure 10:**
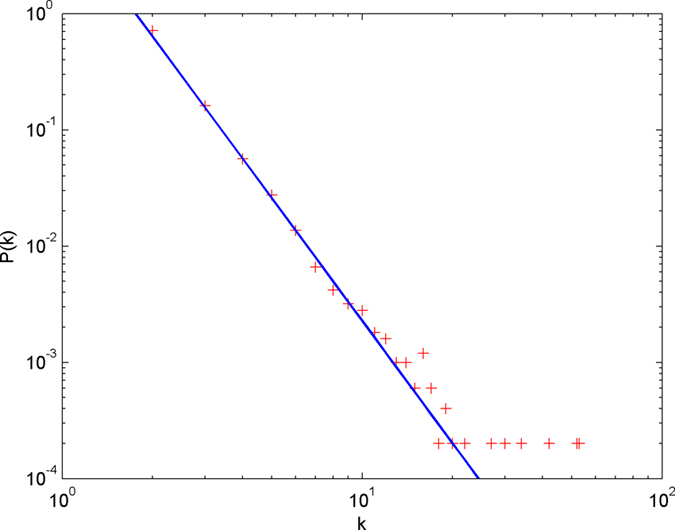
The simulation of the weighted model for node hyperdegree with *m*_1_ = 3, *m*_2_ = 1, *m* = 2, *w*_0_ = 1, *δ* = 0.5. + denotes the simulation result, the line denotes the theoretical prediction.

**Figure 11:**
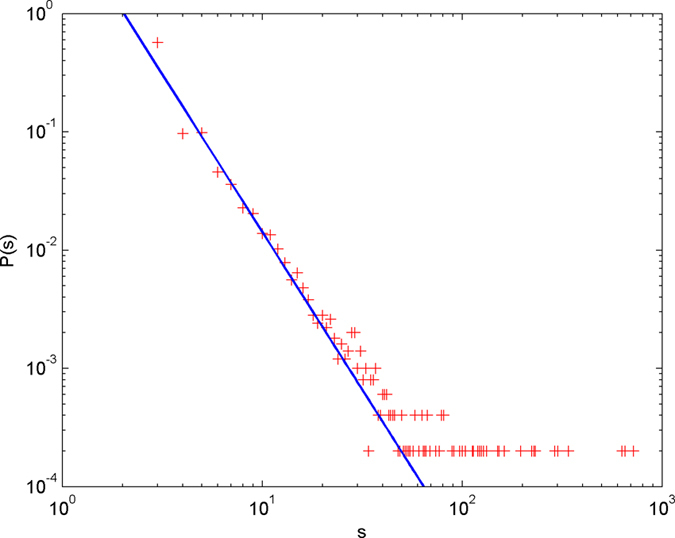
The simulation of the weighted model for node hyperstrength with *m*_1_ = 2, *m*_2_ = 1, *m* = 2, *w*_0_ = 1, *δ* = 1. + denotes the simulation result, the line denotes the theoretical prediction.

**Figure 12:**
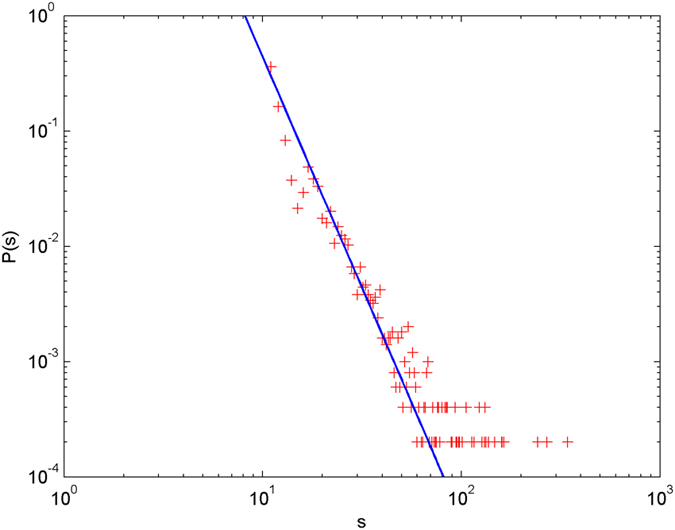
The simulation of the weighted model for node hyperstrength with *m*_1_ = 3, *m*_2_ = 1, *m* = 2, *w*_0_ = 5, *δ* = 1. + denotes the simulation result, the line denotes the theoretical prediction.

**Figure 13:**
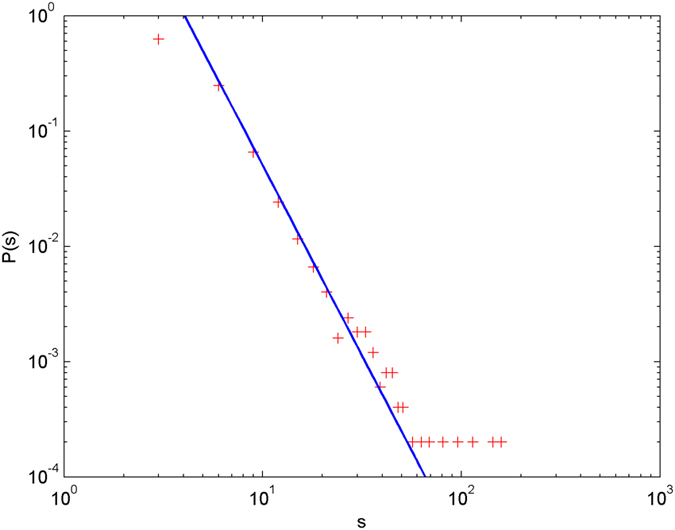
The simulation of the weighted model for node hyperstrength with *m*_1_ = 3, *m*_2_ = 1, *m* = 2, *w*_0_ = 1, *δ* = 0.5. + denotes the simulation result, the line denotes the theoretical prediction.

**Figure 14:**
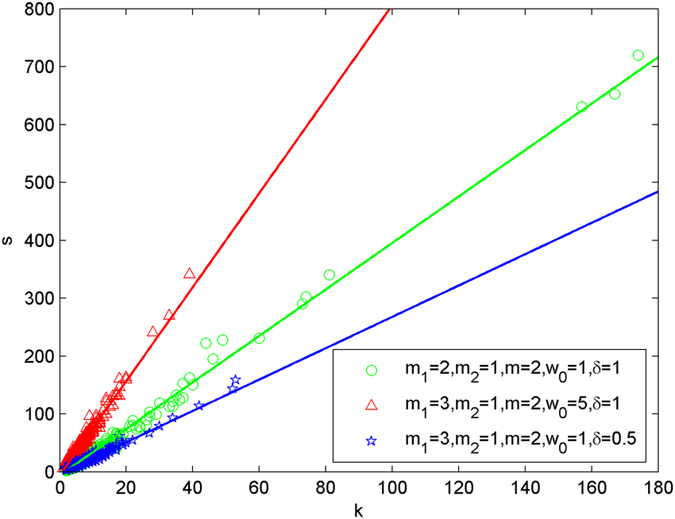
The simulation of the weighted model with the hyperstrength versus hyperdegree for various values.

The following simulations are performed with the scale of N = 5000, and each simulation result is obtained by averaging over 30 independent runs. The simulation results for node hyperdgree and node hyperstrength are shown in Figs 8, 9, 10 and Figs 11, 12, 13 respectively, all in double-logarithmic axis. As the figures show, the simulation results are quite consistent with the theoretical conclusions. The hyperstrength versus hyperdegree for various values are shown in Fig. 14. It can be seen that hyperstrength is positively correlated with hyperdegree.

## Conclusion

This paper proposes a non-uniform model with nonlinear preferential attachment and a weighted model in evolving hypergraphs. In the non-uniform model, at each time step, both the size of new nodes and the randomly selected existing nodes in one hyperedge are random variables. It is clear that non-uniform evolving hypergraphs can better describe real-life systems. We obtain the characteristic equation of hyperdegree and the stationary average hyperdegree distribution of the model. Our theoretical analysis is then verified by numerical simulations. When the model degenerates into a uniform model, the hyperdegree distribution has the form of a generalized power-law, where the power exponent is equal to 
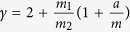
. The weighted model takes into account the fact of topological growth and the dynamic mechanism of the weights. It is found that the weighted model is a special case of the non-uniform model. The study of evolving hypergraphs is necessary for future multidisciplinary research. Applications of these evolving hypergraphs in the study of real-life systems are also worth further investigation. We expect that our results can help accelerate the development of evolving hypergraphs. In this perspective, the models presented in this paper appear as a general starting point for the realistic modeling of weighted evolving hypergraphs.

## Additional Information

**How to cite this article**: Guo, J.-L. *et al*. Non-uniform Evolving Hypergraphs and Weighted Evolving Hypergraphs. *Sci. Rep.* **6**, 36648; doi: 10.1038/srep36648 (2016).

**Publisher’s note**: Springer Nature remains neutral with regard to jurisdictional claims in published maps and institutional affiliations.

## Figures and Tables

**Figure 3:**
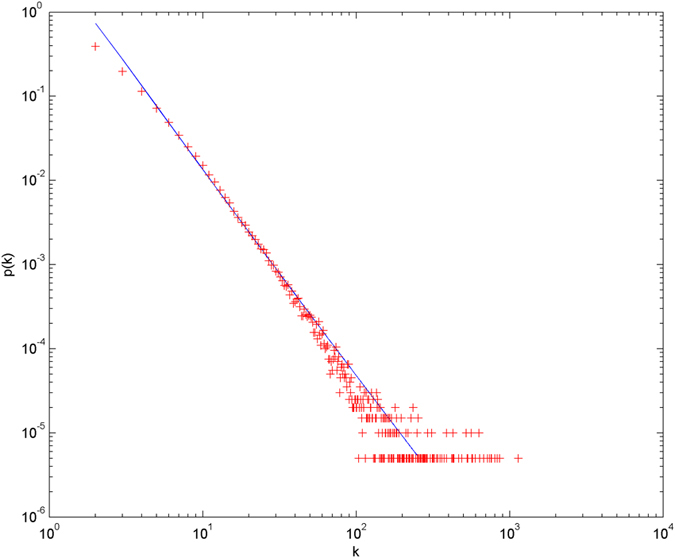
The simulation of the non-uniform model. *α* = 1, 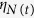 is randomly selected from 1 ~ 2, 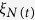 is randomly selected from 1 ~ 4. + denotes the simulation result, the line denotes the theoretical prediction.

**Figure 4:**
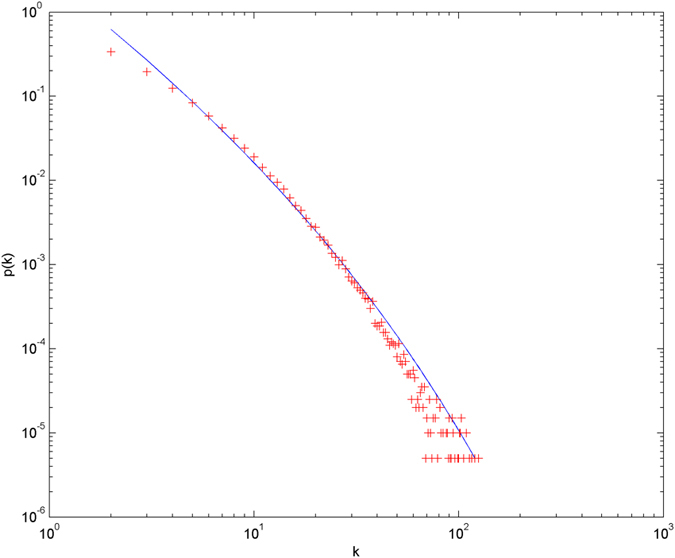
The simulation of the non-uniform model. 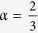
, 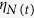 is randomly selected from 1 ~ 3, 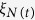 is randomly selected from 1 ~ 5. + denotes the simulation result, the line denotes the theoretical prediction.

**Figure 5:**
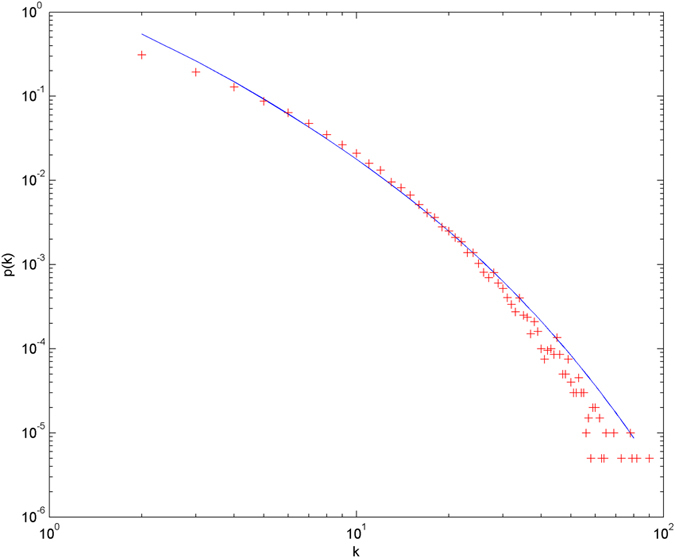
The simulation of the non-uniform model. 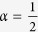
, 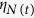 is randomly selected from 1 ~ 3, 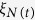 is randomly selected from 1 ~ 5. + denotes the simulation result, the line denotes the theoretical prediction.
